# Tau Imaging in Neurodegenerative Diseases Using Positron Emission Tomography

**DOI:** 10.1007/s11910-019-0962-7

**Published:** 2019-06-06

**Authors:** Yi Ting Wang, Paul Edison

**Affiliations:** 1 Neurology Imaging Unit, Division of Brain Sciences, Department of Medicine, Imperial College London, 1st Floor B Block, Hammersmith Hospital Campus, Du Cane Road, London, W12 0NN UK; 2 Cardiff University, Cardiff, CF10 3AT UK

**Keywords:** Positron emission tomography, Tau, Neurofibrillary tangles, Dementia, Neurodegenerative diseases, Neurodegeneration

## Abstract

**Purpose of Review:**

Abnormal accumulation of tau protein is the main hallmark of tauopathies and is closely associated with neurodegeneration and cognitive impairment, whereas the advance in PET imaging provides a non-invasive detection of tau inclusions in the brain. In this review, we discuss the potential of PET imaging as a biomarker in tauopathies, the latest development of novel tau tracers with new clinical information that has been disclosed, and the opportunities for improving diagnosis and designing clinical trials in the future.

**Recent Findings:**

In recent years, several first-generation tau PET tracers including [^11^C]PBB3, [^18^F]THK-5117, [^18^F]THK-5351 and [^18^F]AV-1451 have been developed and succeeded in imaging neurofibrillary pathology in vivo. Due to the common off-target binding and subcortical white matter uptake seen in the first-generation tracers, several research institutes and pharmaceutical companies have been working on developing second-generation tau PET tracers which exhibit higher binding affinity and selectivity.

**Summary:**

Tau PET imaging is promising to serve as a biomarker to support differential diagnosis and monitor disease progression in many neurodegenerative diseases.

## Introduction

Neurofibrillary tangles (NFTs) are one of the primary hallmarks of Alzheimer’s disease (AD), along with amyloid plaques, cerebral amyloid angiopathy and glia activation [1]. NFTs are aggregates of hyperphosphorylated tau protein and they form before the clinical symptoms of AD manifest. The amount of NFTs was reported to be tightly linked to the severity of AD-type dementia, suggesting that they are better correlated with neuronal dysfunction [2]. Cortical density of hyperphosphorylated tau tangles in AD postmortem brain also correlates with pre-morbid cognitive dysfunction and neuronal loss [3]. It is important to note that besides AD, accumulation of tau protein is observed in numerous other diseases known as tauopathies. Tauopathies is the term used to describe a group of neurodegenerative conditions characterised by the abnormal accumulation of tau aggregates in the brain. Along with AD and some variants of frontotemporal lobe degeneration (FTLD), other tauopathies include Pick’s disease, frontotemporal dementia with parkinsonism linked to chromosome-17 (FTDP-17), progressive supranuclear palsy (PSP), corticobasal degeneration (CBD) and chronic traumatic encephalopathy (CTE) [4]. Although all these disorders share tau immunoreactivity in postmortem analysis, they can be composed of different tau isoforms and show distinct histopathological differences [5].

**Fig. 1:**
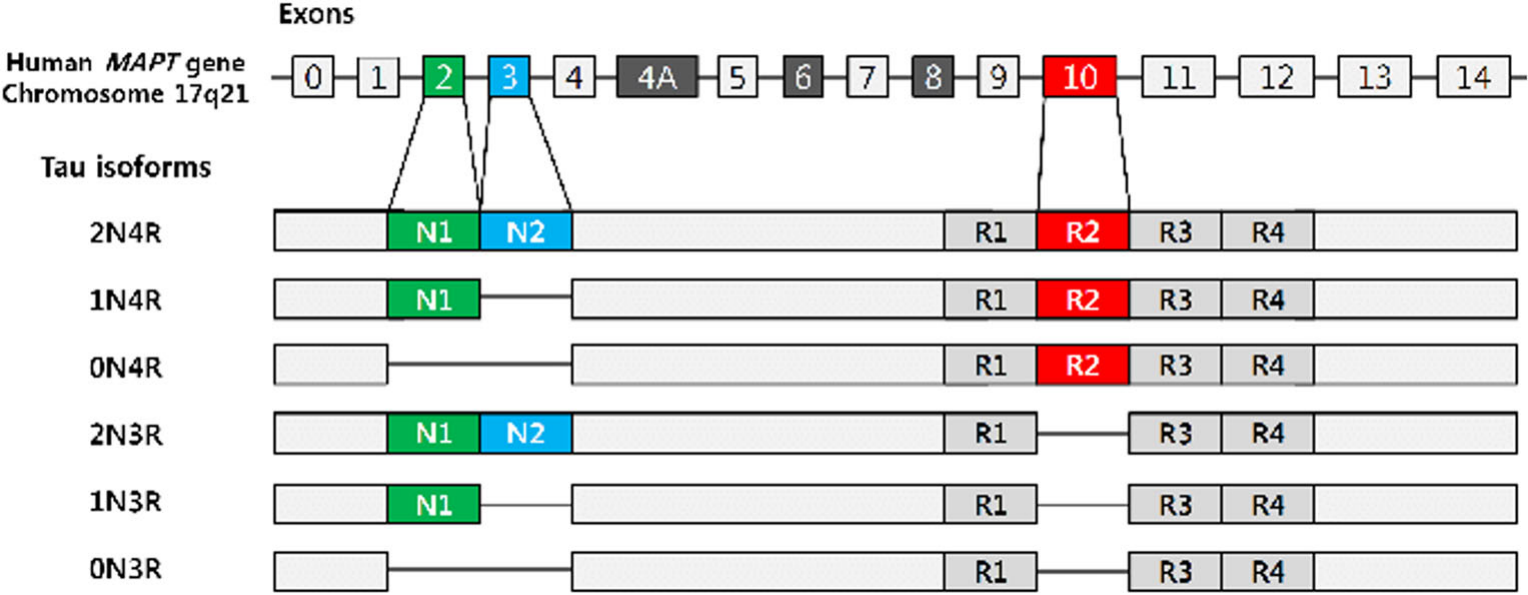
Tau protein isoforms in the human CNS. Six tau isoforms are present in the brain. N1 (green) and N2 (blue) are produced through the alternative splicing of exons 2 and 3 respectively. Exon 10 encodes the second aspect of the microtubule-binding repeat domain, R2 (red). Depending on the presence of the R2 domain, tau proteins are termed either 3R-tau or 4R-tau. (Figure used with permission from BMB Rep. Park SA, Ahn SI, Gallo J-M. Tau mis-splicing in the pathogenesis of neurodegenerative disorders. BMB Rep. 2016;49:405–413)

There are six isoforms of tau expressing in adult human central nervous system resulting from the alternative splicing of pre-mRNA generated from microtubule-associated protein tau (*MAPT*) gene. These tau isoforms can contain either three or four microtubule–binding repeats, termed 3R-tau or 4R-tau respectively. Tau protein isoforms in the human brain are demonstrated in Fig. 1 [6].

Under normal physiological condition, 3R-tau and 4R-tau are expressed at similar level; however, in the brains in several tauopathies, the 3R-tau/4R-tau ratio is altered [7]. Comparison of the tau aggregates in different neurodegenerative disorders reveals high diversity in both the degree of phosphorylation and the content of the different tau isoforms. This could possibly explain the variation in tau inclusion morphology and cellular specificity. Tauopathies, as a result, may be subdivided into disorders with inclusions made predominantly of 3R- or 4R-tau or a combination of both [8]. A brief molecular pathological classification of tauopathies is summarised in Table 1.

**Table 1:** Pathological classification of Tauopathies

Predominant tau isoforms	3R-tau	4R-tau	3R- & 4R-tau
Disorders	• Pick’s disease (PiD)	• PSP	• NFT dementia (including AD)
	• FTLD-*MAPT*	• CBD	• PART
	• FTDP-17 with 3R-tau	• AGD	• CTE
		• GGT	• FTLD-*MAPT*
		• FTLD-*MAPT*	• FTDP-17 with 3R- & 4R-tau
		• FTDP-17 with 4R-tau	

3R-tau: tau protein isoform with 3 repeats in the microtubule-binding domain; 4R-tau: tau protein isoform with 4 repeats in the microtubule-binding domain; 3R- & 4R-tau: mixed 3 and 4 repeat tau protein isoforms

*PSP* progressive supranuclear paralysis, *CBD* corticobasal degeneration, *AGD* argyrophilic grain disease, *GGT* globular glial tauopathy, *CTE* chronic traumatic encephalopathy, *PART* primary age–related tauopathy, *FTLD-MAPT* Frontotemporal lobar degeneration associated with mutations in *MAPT*

Among the main pathophysiological hallmarks of AD, NFTs have a stereotypical spatiotemporal progression that correlates better with the severity of the cognitive decline seen in the disease compared to Aβ plaques [1]. NFTs start in the allocortex of the medial temporal lobe (entorhinal cortex and hippocampus) and spreads to the associative neocortex, relatively sparing the primary sensory, motor and visual areas. In Braak staging of AD, NFTs first appear in the perirhinal region along with the entorhinal cortex (stage I), followed by the CA1 region of the hippocampus (stage II). NFTs later develop and accumulate in the limbic structures such as the hippocampal formation (stage III) and the amygdala, thalamus and claustrum (stage IV). Eventually, NFTs spread to all neocortical areas, with the association areas being affected prior and more severely (stage V) than the primary sensory, motor and visual areas (stage VI) [9]. Thus, biomarkers for tau pathology are considered promising and essential to the research criteria in the pathological diagnosis of AD.

### PET Imaging as a Biomarker in Tauopathies

The advance in molecular imaging has shed light on the use of imaging biomarker in the diagnosis of AD as well as many other neurodegenerative diseases. Positron emission tomography (PET) is a non-invasive diagnostic imaging modality utilising isotope-labelled molecular probes that bind to molecules with both high specificity and affinity. In recent years, several PET tracers targeting abnormal conformations of the tau protein have been developed, which allowed researchers to visualise tau aggregate in vivo [10]. As tau is a complex protein with multiple isoforms and post-translational modifications, tau PET tracers may bind to specific or multiple isoforms. Moreover, since tau is an intracellular protein, these radioligands will need to possess the ability to cross the plasma cell membranes as well as the blood-brain barrier (BBB). Tau aggregates are coexistent with Aβ plaques which both share β-sheet structure in human AD, even though it is 5 to 20 times lower concentrations than Aβ plaques [11]. This suggests that the radioligands need to be highly selective with at least 10-fold higher binding affinity for tau compared with Aβ [3]. To date, several PET ligands including [^11^C]PBB3, [^18^F]THK5105, [^18^F]THK5117, [^18^F]THK5351 [^18^F]T807 and [^18^F]T808 have been tested to image tau neurofibrillary tangle deposition in living AD patient brains [12–14, 15••]. Some of these tau PET tracers are considered superior to others and are now available for clinical assessment of patients with various tauopathies, including AD, as well as in healthy subjects. They provide the opportunity of in vivo topographical distribution and quantification of tau aggregates in the early phases of neurodegenerative diseases, in parallel with clinical and cognitive assessments. As such, tau imaging is considered of key importance for progress toward earlier and more accurate diagnosis of tauopathies as well as for the tracking of disease progression, monitoring therapeutic interventions and drug development.

In this review, we examine both preclinical and clinical PET studies of the first-generation tau PET tracers, discussing the promising usefulness and the difficult challenges, as well as the opportunities for tau PET imaging used to improve diagnosis and help designing clinical trials. We further demonstrate new clinical information that has been uncovered regarding newly developed novel second–generation tau tracers.

### First-Generation Tau Tracers

#### [^18^F]FDDNP

2-(1-{6-[(2-[fluorine-18]Fluoroethyl)(methyl)amino]-2-naphthyl}-ethylidene)malononitrile (FDDNP) is the first PET tracer to visualise Alzheimer’s disease pathology in living humans [16]. FDDNP is not an exclusive tau marker. In fact, it binds to both neurofibrillary tangles and amyloid plaques in the brains. In the first longitudinal PET study comparing the tracer binding of [^11^C]PIB and [^18^F]FDDNP in AD, mild cognitive impairment (MCI) patients and healthy controls, [^18^F]FDDNP successfully discriminated between AD and healthy controls yet with a ninefold lower specific binding signal in comparison with [^11^C]PIB, which was thought to be due to both amyloid and tau [17]. Interestingly, the development of FDDNP opened the door for both amyloid and tau PET imaging in AD, as well as other tauopathies. It was an important milestone since postmortem histopathological studies have consistently demonstrated that NFTs are better index of disease severity and progression, compared to Aβ. In addition, high clinical trial failure rate of disease-modifying drugs suggested that some of the basic assumptions of AD aetiology warrant reassessment and redirection [16].

**Fig. 2:**
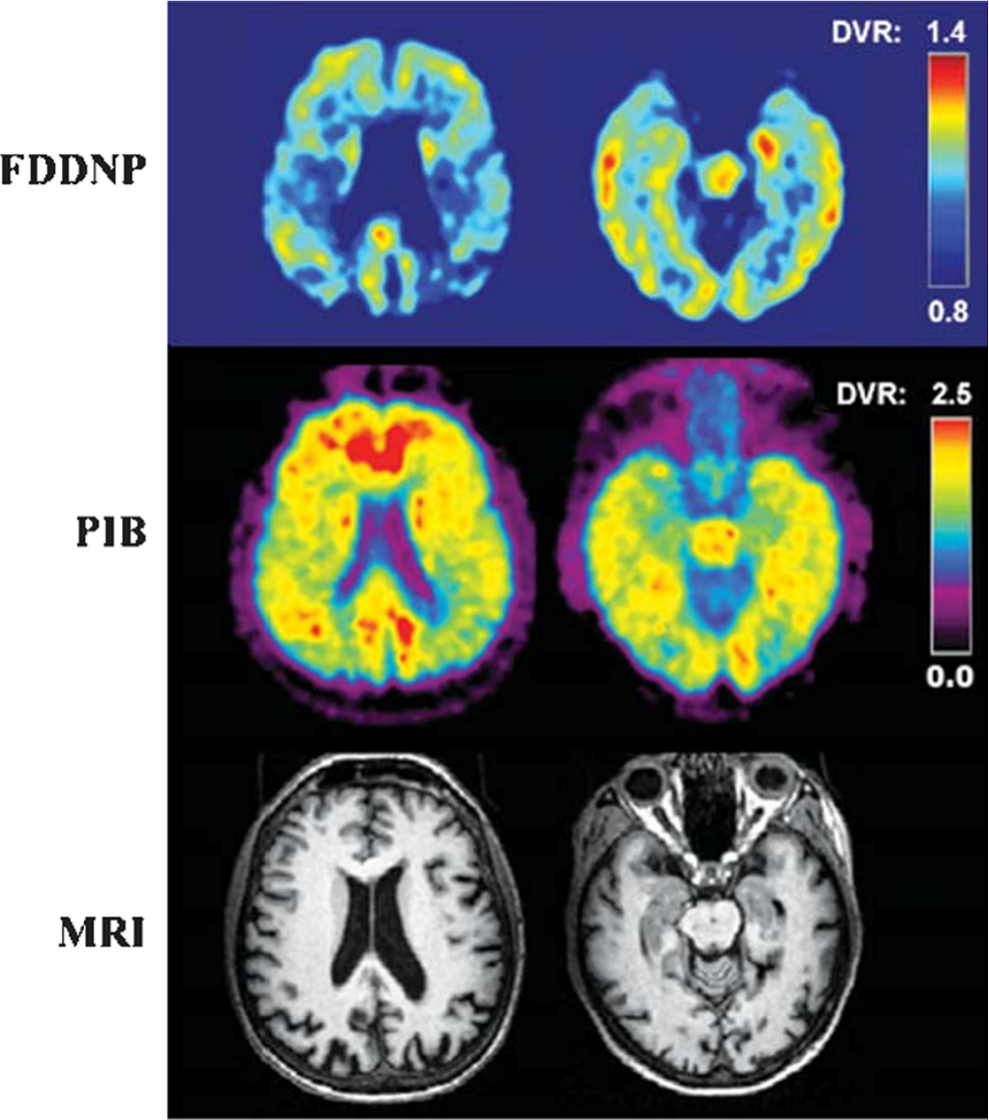
[^18^F]FDDNP-PET images. Co-registered FDDNP-PET, PIB-PET and MRI scans of a patient with Alzheimer’s disease. Images in the left column: parietal lobe; images in the right column: temporal lobe. [16]. (Reprinted from J. Alzheimers Dis;26. Shin J, Kepe V, Barrio JR, Small GW. The merits of FDDNP-PET imaging in Alzheimer’s disease., Suppl 3:135–145. Copyright (2011), with permission from IOS Press”.) The publication is available at IOS Press through 10.3233/JAD-2011-0008

Figure 2 demonstrates PET images of [^18^F]FDDNP used in an AD patient.

#### [^11^C]PBB3

Among the first generation of tau PET tracers, ^11^C-pyridinyl-butadienyl-benzothiazole 3 ([^11^C]PBB3) was reported to detect a broad range of tau deposits. [^11^C]PBB3, developed by Higuchi and his colleagues, is a clinically used PET tracer allowed in vivo detection of tau inclusions in Alzheimer’s disease as well as non-AD tauopathies in the human brain. While the signal of [^11^C]Pittsburgh compound B (PiB), one of the most commonly used PET tracer for detecting amyloid plaques, barely changed after the clinical onset of AD, [^11^C]PBB3 successfully demonstrated spreading of brain tau pathology in transition from normal ageing to moderate AD, suggesting the usefulness of tau PET imaging as an objective index of disease progression [18]. This finding is in line with the notion that tau lesions are more tightly associated with neuronal dysfunction and disease progression than are Aβ plaques.

**Fig. 3:**
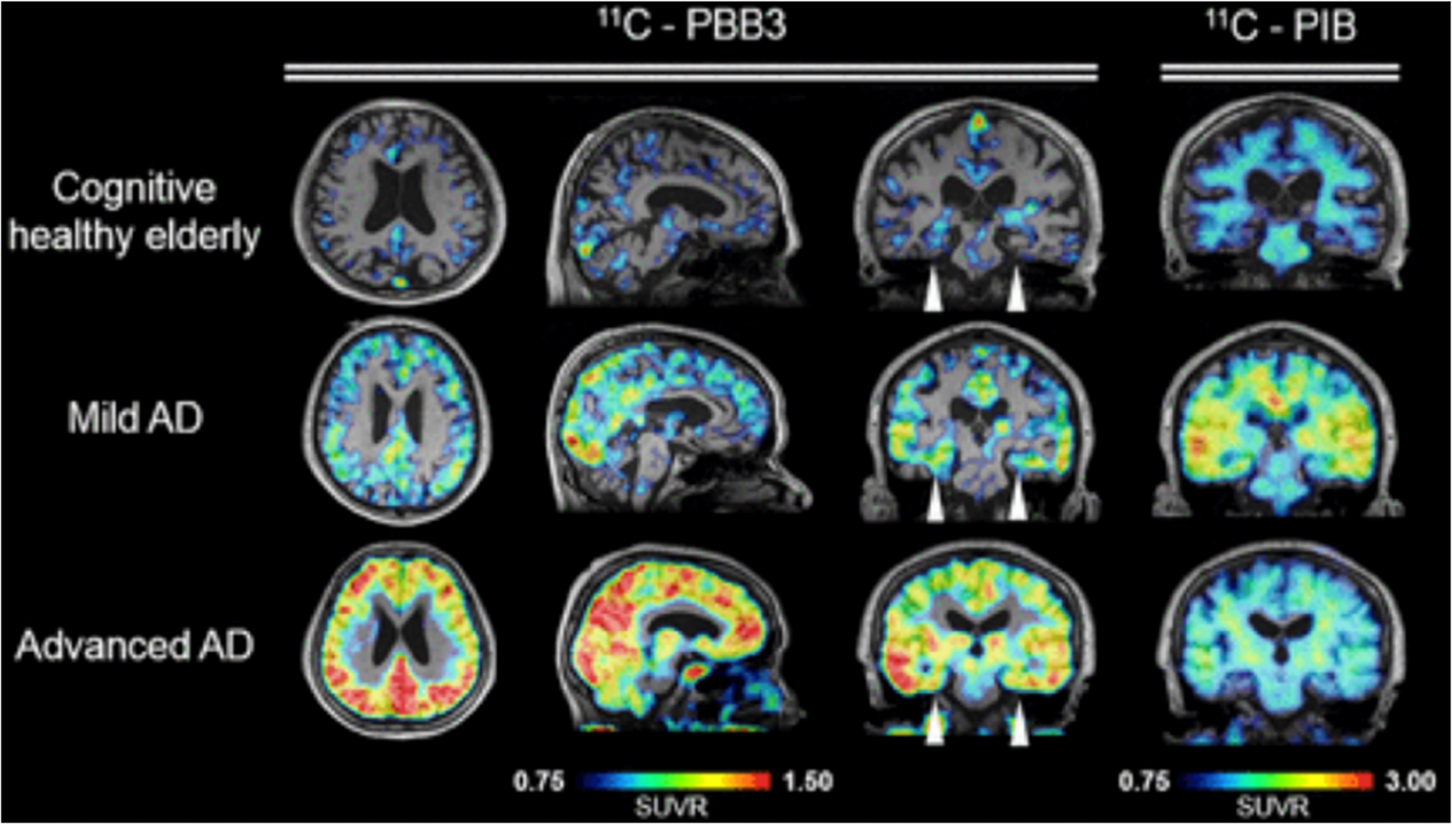
[^11^C]PBB3 PET images. [^11^C]PBB3 parametric standardised uptake value ratio (SUVR) images compared with [^11^C]PiB. [^11^C]PBB3 shows tracer uptake in cortical areas in advanced AD patient. This research was originally published in *JNM*. Shah M, Catafau AM. Molecular Imaging Insights into Neurodegeneration: Focus on Tau PET Radiotracers. J. Nucl. Med. 2014;55:871–874. © SNMMI [21]

In clinical PET human studies, [^11^C]PBB3 clearly differentiated AD brains from healthy control brains and tracer retention in the hippocampus confirmed the binding ability to NFTs [12]. In AD patients, specific [^11^C]PBB3 binding was observed in the CA1 and subiculum regions in the hippocampus, where high density of fibrillar tau aggregates exist [12]. High binding of [^11^C]PBB3 was also detected in the medial temporal lobes, the precuneus and the frontal cortex. More importantly, the tracer retention was well correlated with cognitive decline and grey matter atrophy [19••]. Besides for its utility in Alzheimer’s disease, in vivo PET data provided evidence that [^11^C]PBB3 was capable of detecting tau aggregates in patients with non-AD dementias as exemplified by CBD as well [12]. Another study reported significant [^11^C]PBB3 binding to tau deposits in the basal ganglia of a CBD case [20]. More studies of [^11^C]PBB3 will validate the clinical usefulness of this tracer in various types of tauopathies. An example of [^11^C]PBB3 images in AD patient is showed in Fig. 3.

#### Quinoline Derivatives (THK Compounds)

Novel quinoline derivatives were initially identified as candidates for tau PET tracer by the screening of over 2000 small molecules [22]. In vitro studies showed that three quinolone–derived tracers, [^18^F]THK-523, [^18^F]THK-5105 and [^18^F]THK-5117, demonstrated selective and high binding affinity to tau over Aβ on AD brain sections [23–25]. However, [^18^F]THK-523 failed to clearly visualise tau deposits in the human brain in vivo [26]. By contrast, [^18^F]THK-5105 successfully demonstrated radiotracer retention in sites susceptible to tau deposition in the AD brain [27]. Recent PET studies demonstrated increased [^18^F]THK5105 and [^18^F]THK5117 tracer uptake in common sites of tau pathology in AD and its association with clinical severity of dementia [27, 28]. It is important to mention that [^18^F]THK-5117 PET showed higher signal-to-background ratio and better pharmacokinetics compared to [^18^F]THK-5105 [21]. Despite the exciting results showed by many studies, these THK compounds, like amyloid PET tracers, showed high nonspecific retention in the subcortical white matter. Signal from white matter binding could possibly obscure visual interpretation of PET images and decrease detection sensitivity for early tau pathology in the prodromal AD.

To minimise nonspecific tracer retention in the white matter and increase the signal-to-background ratio of PET images, Harada and colleagues replaced a benzene ring of [^18^F]THK5117 with pyridine and developed a novel tau PET tracer, [^18^F]THK5351. [^18^F]THK5351 is a single S-enantiomer, which was expected to improve the pharmacokinetics of arylquinoline derivatives [15••]. The group reported a higher binding affinity for hippocampal homogenates from AD brains and faster dissociation from white matter tissue of [^18^F]THK5351 compared to [^18^F]THK5117. The level of tracer binding is still well correlated with the amount of tau deposits in human brain samples. More importantly, [^18^F]THK5351 selectively bound to NFTs with a higher signal-to-background ratio than [^18^F]THK5117 did. First human PET studies showed that [^18^F]THK5351 has faster kinetics, higher contrast and lower retention in the subcortical white matter, suggesting that it is a useful PET tracer for the early detection of neurofibrillary pathology in AD patients [15••]. However, recent studies have demonstrated that monoamine oxidase inhibitor can reduce [18F]THK5351 uptake in the human brain.

**Fig. 4:**
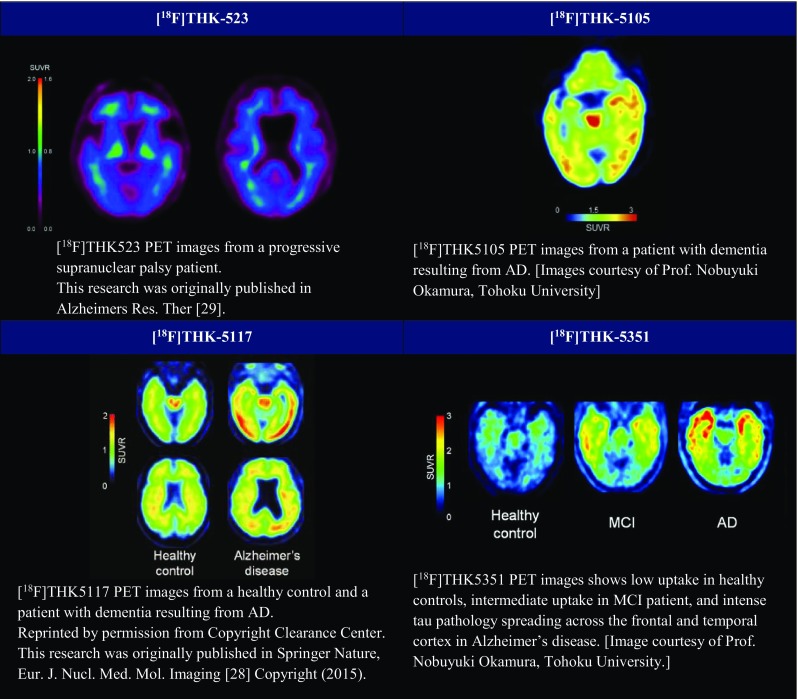
PET images of THK compounds. [^18^F]AV-1451 (T807). [^18^F]AV-1451 (flortaucipir, T807) exhibited strong and selective binding affinity to PHF-tau on AD brain [13, 30]. With favourable pharmacokinetics and a binding pattern consistent with the Braak staging, [^18^F]AV-1451 is considered as the most promising PET candidates for tau imaging [21]. Clinical [^18^F]AV-1451 PET scans are demonstrated in Fig. 5. (THK-523 This research was originally published in Alzheimers Res. Ther) [29] (THK-5105 and THK-5351 Images courtesy of Prof. Nobuyuki Okamura, Tohoku University) (THK-5117 Reprinted by permission from Copyright Clearance Center. This research was originally published in Springer Nature, Eur. J. Nucl. Med. Mol. Imaging [28] Copyright (2015)

Figures of these THK compounds are illustrated in Fig. 4.

The first human PET study successfully demonstrated [^18^F]AV-1451 retention in the frequent areas of PHF-tau in the AD brain [13]. In addition, the tracer retention was associated with increased disease severity, supported by the highly elevated and extensive [^18^F]AV-1451 retention in severe AD case than in MCI and mild AD cases. Unlike most ^18^F-labelled amyloid PET tracers, [^18^F]AV-1451 showed very low non-specific binding to the white matter, which may improve the grey-to-white matter contrast in the brain. However, it is worth noticing that AV1451 showed substantial variability in healthy controls, especially subjects younger than 40. Suzanne Baker and colleagues found that non-specific binding of [^18^F]-AV1451 in the brain reflects both age-related (putamen) and non-age-related pattern (white matter/thalamus). The results suggested that these factors may reduce signal to noise at low levels of tracer binding [31••]. Interestingly, an in vivo study reported that [^18^F]AV-1451 showed higher level of binding in microtubule-associated protein tau (*MAPT*) mutation carriers who harbour mutations that are more likely to produce AD-like tau pathology. This finding further supported the notion that [^18^F]AV-1451 has specific binding properties for AD-like tau pathology. Most excitingly, [^18^F]AV-1451 has just been announced to meet its two primary endpoints, defined as predicting brain tau pathology and predicting Alzheimer’s disease diagnosis, in a phase III study (A16 trial). Primary outcomes showed a pattern of tracer uptake that corresponded to NFT accumulation consistent with Braak stage V/VI, and a pattern consistent with high levels of AD neuropathologic change as defined by National Institute on Aging-Alzheimer’s Association (NIA-AA) criteria. However, the data would not be able to prove the reliability of [^18^F]AV-1451 in people at earlier Braak stages for example stages III and IV, which are clinically meaningful. Further research is needed for the validation of the tracer.

In a recent study aiming for modelling amyloid, tau and cortical thickness changes across the Alzheimer’s disease spectrum, Jungho Cha et al. found that AV1451 appeared to increase after PiB reached a plateau, but preceded changes in cortical thickness [32]. These results were consistent with the notion that AD is an amyloid-facilitated tauopathy and in turn leads to atrophy of neurons. In another study examining effects of APOE ε4 on tau pathology in AD, Niklas Mattsson and colleagues found that APOE ε4-negative AD patients showed significantly greater tau load and reduced cortical thickness specifically in the parietal cortex compared to their APOE ε4-positive counterparts [33]. A longitudinal tau PET study running in ageing and AD subjects by Clifford Jack et al. showed that the rate measurements based on Braak topographic staging of NFTs or voxel-wise approaches may not necessarily provide substantially more information than simple meta-ROI rate measurements. They further suggested that tau PET standardised uptake value ratio (SUVRs) measures would be an efficient outcome measurement in disease-modifying clinical trials [34••]. Another recent study by Tharick Pascoal et al. examined the voxel-wise sensitivity, specificity, area under the curve (AUC) and thresholds of neurofibrillary tangle deposition in AD using [^18^F]AV1451 and [^18^F]MK6240. Their findings indicated that the regional thresholds of tau PET ligands have the potential to be used in clinical trials for the enrolment of individuals with tau abnormalities [35]. It is very important to note that although tau has been indicated to strongly associate with cognition, considerable overlap of tau PET measurements across cognitively normal and AD subjects has been observed. Susan Landau and colleagues found that AV1451 SUVRs of one third to one half of amyloid-positive MCI and AD patients overlapped with amyloid-negative healthy controls. This finding poses a challenge for using tau as a biomarker for cognitive impairment.

[^18^F]T808 showed a high level of binding affinity and good selectivity for tau aggregates over amyloid β plaques in in vitro assays as well as rapid uptake and washout in rodent brains. Further preclinical in vivo studies suggested that [^18^F]T808 possesses suitable properties and characteristics to be a specific and selective PET probe for imaging of paired helical filament tau in human brains [36]. More rapid tracer distribution throughout the brain and more rapid clearance from normal brain tissue of [^18^F]T808 compared to [^18^F]AV-1451 were observed in human clinical studies [14]. However, substantial defluorination has been observed with [^18^F]T808, and thus it is not taken forward [11].

### Current Challenges in Developing Tau Tracers

The development of tau imaging radioligands has enabled visualisation of tau depositions in tauopathy patients, but the modes of their binding to different tau strains still remains unclear. In the development of PET radioligand for tau imaging, several aspects have made it challenging.

First, the candidate PET ligands were designed to target β-pleated sheets, a protein structural motif that is shared by both tau and Aβ, thereby raising the possibility of cross-reaction [18]. The fact that tau aggregates are coexistent with Aβ plaques with 5 to 20 times lower concentrations than Aβ plaques made it even more difficult to develop a highly selective tau PET imaging tracers [11]. Secondly, the complexity of developing a radioligand targeting tau protein is increased because of its intracellular location [37••], which means the ligands need to be able to pass through the plasma membrane as well as the blood–brain barrier in order to reach tau protein [18]. Another critical challenge is primarily due to the nature of tau protein in human brains. Through alternative splicing of the *MAPT* gene, six isoforms of tau are expressed in the adult human central nervous system, giving rise to two sets of isoforms: 3R-tau and 4R-tau. Diverse neurodegenerative disorders are characterised by deposition of tau fibrils composed of conformers (i.e., strains) unique to each illness. Under pathological circumstances, several tauopathies express different isoform ratios with diverse morphologies. These structural differences lead to the difficulty of developing a tau-specific tracer, with similar affinity for every phenotype [38]. Furthermore, tau is subjected to many posttranslational modifications, which may result in conformational changes in the aggregates, potentially leading to different binding affinities of tau ligands [39]. Last is the “off-target” binding of the tracers. It has become clear that these tracers detected the distribution of not only tau but also other proteins in the brain, which might possibly due to the structural motifs [40]. For example, both [^18^F]AV-1451 and [^18^F]THK5351 have been found to also bind to monoamine oxidases (MAO) [41•, 42]. Moreover, [^18^F]AV-1451 is well known to bind to calcifications, iron, melanin and blood vessels [43].

### Comparison of First-Generation Tau Tracers

Diverse neurodegenerative disorders are characterised by the abnormal accumulation of tau fibrils composed of different strains unique to each illness. Several PET tracers have been developed for visualisation of tau deposition under heterogeneous tau pathology, but the modes of their binding to different tau strains remain unclear [44•, 45••]. Head-to-head comparison of different tau tracers is commonly used for researchers to evaluate the pharmacokinetics of different tracers in terms of their uptake, distribution, clearance and metabolism. This provides crucial information for the selection of PET imaging ligands capable of binding to one or more tau fibril strains in different tauopathies.

One in vivo study evaluated the binding of [^11^C]THK5351 and [^11^C]PBB3 in a head-to-head multimodal design. The findings indicated different molecular targets for these tracers. While [^11^C]PBB3 appeared to preferentially bind to tau deposits with a close spatial relationship to Aβ, the binding pattern of [^11^C]THK5351 fitted the expected distribution of tau pathology in Alzheimer’s disease better and was more closely related to downstream disease markers [46]. [^11^C]PBB3 and [^18^F]AV-1451 were also compared in an in vitro study. Results indicated distinct selectivity of [^11^C]PBB3 compared to [^18^F]AV-1451 for diverse tau fibril strains. This highlighted the more robust ability of [^11^C]PBB3 to capture wide-range tau pathologies [47•]. In a preclinical study using mouse model of tau pathology, tracer uptake in the brainstem of [^18^F]AV-1451 showed to be moderately superior to [^18^F]THK5117 regarding sensitivity for preclinical tau imaging. Another study compared [^18^F]AV-1451 and [^18^F]THK5351 in Alzheimer’s disease and frontotemporal dementia cases. Although AV-1451 and THK5351 uptakes were highly correlated, cortical uptake of AV-1451 was more striking in Alzheimer’s disease, while cortical uptake of THK5351 was more prominent in frontotemporal dementia. THK5351 showed higher off-target binding than AV-1451 in the white matter, midbrain, thalamus and basal ganglia. The results indicated that AV-1451 is more sensitive and specific to Alzheimer’s disease type tau, while THK5351 may mirror general non-specific neurodegeneration [48].

A brief summary of first-generation tau tracers is listed in Table 2. Some recent clinical study using different first-generation tau tracers are summarised in Table 3.

**Table 2:** Comparison of first-generation tau PET tracers

Tracer	[^11^C]PBB3	[^18^F]THK-5105	[^18^F]THK-5117	[^18^F]THK-5351	[^18^F]AV-1451
Affinity (tau isoforms)	3R & 4R (can detect non-AD tau)	3R & 4R	3R & 4R	3R & 4R	3R & 4R (low affinity for non-AD tau)
Selectivity (tau-over-Aβ ratio)	40- to 50-fold	25-fold	No Aβ binding	No Aβ binding	> 27-fold
Tracer spatial distribution in human brain	Temporal cortices (especially MTL including hippocampal formation)	Temporal cortices (especially inferior & medial part)		Temporal cortices	Temporal cortices
					Parietal cortices
		Parietal cortices			Frontal cortices
		Posterior cingulate			Hippocampal formation
Advantages	-Capable of capturing the progression of AD	-Correlated well with cognitive parameters, hippocampal and whole brain grey matter volumes	-Faster pharmacokinetics	-Higher binding affinity for hippocampal homogenates and faster dissociation from white matter tissue than THK-5117	-High levels of PHF-tau binding affinity, selectivity, specificity and lipophilicity
	-Affinity for different tau isoforms		-Higher signal-to-background ratios than THK-5105		
					-Desirable pharmacokinetic
	-Reduced exposure to radioactivity				
					-Little white matter binding
					-Non-detectable/minimal binding toward off-target proteins such as MAO
				-Higher signal-to-background ratio than THK-5105 and THK-5117	
Challenges	-Off-target binding in dural venous sinuses	-Off-target binding in brainstem, thalamus, and subcortical white matter	-Off-target binding in subcortical white matter	-Off-target binding in basal ganglia	
	-Short radioactive half-life of [^11^C]				
		-High background signals in grey matter			
References	[12, 18, 19••]	[25, 27]	[28]	[15••]	[13, 30]

**Table 3:** Highlight of recent clinical human PET study

Subjects	Major findings	References
9 MCI patients (prodromal AD)	-[^11^C]PBB3 preferentially bind to tau deposits with a close spatial relationship to Aβ	[46]
	-[^11^C]THK5351 presented the distribution of tau pathology in AD better and was more closely related to downstream disease markers	
3 AD patients and 3 HCs	[^11^C]PBB3 tracer distribution was consistent with the spreading of tau pathology with AD progression	[12]
2 AD patients, 4 FTD patients (frontotemporal dementia) and 2 HCs	-[^18^F]AV-1451 is more sensitive and specific to Alzheimer’s disease type tau and shows lower off-target binding	[48]
	[^18^F]THK5351 may better present non-specific neurodegeneration	
69 healthy controls (PIB negative)	Explained [^18^F]AV-1451 variability in healthy controls across the lifespan.	[31]
6 AD patients, 3 PSP patients, 2 CBS patients and 4 HCs	Described the kinetics of [^18^F]AV-1451, the optimal scanning time and the reference region for SUVR calculation.	[49]
3 subjects carrying the *MAPT* R406W mutation	[^18^F]AV-1451 PET can be used to accurately quantify in vivo the regional distribution of hyperphosphorylated tau protein.	[50]
20 EOAD patients, 21 LOAD patients, 3 prodromal EOAD 13 prodromal LOAD and 30 HCs	Described the difference in [^18^F]AV-1451 tracer retention in early- and late-onset Alzheimer’s disease.	[51]
39 AD patients, 14 prodromal AD and 30 HCs	Elucidated the relationship of [^18^F]AV-1451 tracer retention to tau in cerebrospinal fluid.	[52•]
11 PSP patients and 11 HCs	Characterised the tracer uptake of [^18^F]AV-1451 in progressive supranuclear palsy.	[53•]
		[54]
One 71-year-old male subject		
31 AD patients, 11 PSP patients, 8 CBS patients and 17 HCs	Characterised the tracer uptake of [^18^F]AV-1451 in corticobasal syndrome.	[55•]
17 AD patients and 95 HCs	Regional thresholds of [^18^F]AV-1451 have the potential to be used in clinical trials for the enrolment of individuals with tau abnormalities.	[35]
59 cognitively unimpaired with normal amyloid (CUA-)	-Rate measurements based on granular Braak–like topographic staging or voxel-wise approaches may not provide significantly more information than simple meta-ROI rate measurements.	[34••]
37 cognitively unimpaired with abnormal amyloid (CUA+)		
30 cognitively impaired with amnestic phenotype and abnormal amyloid (CIA+)	-Tau PET SUVR measures should be an efficient outcome measure in disease-modifying clinical trials.	

### Second-Generation Tau Tracers

Imaging agents capable of quantifying the brain’s tau aggregates allow a more precise staging of Alzheimer’s disease. Since first-generation tau PET ligands bind predominantly to AD-typical 3R-/4R-tau isoforms and exhibit off-target binding as well as subcortical white matter uptake, several research institutes and pharmaceutical companies have been trying to improve the binding selectivity and pharmacokinetics of tau PET tracers. These second-generation tau PET ligands appear to bind to comparable binding sites but showed less brain off-target binding. Chemical structures of first- and second-generation tau PET tracers are illustrated in Table 4. ^18^F-labelled PBB derivatives, APN-1607 ([^18^F]PM-PBB3) showed a greater signal-to-background ratio and less off-target signals in the basal ganglia than [^11^C]PBB3. [^18^F]GTP1, another second-generation tau tracer, was showed to correlate with AD pathology. A clinical PET study of [^18^F]GTP1 successfully prevented tracer accumulation in the skull and clearly differentiated AD patients from healthy control subjects [56].

**Table 4:**
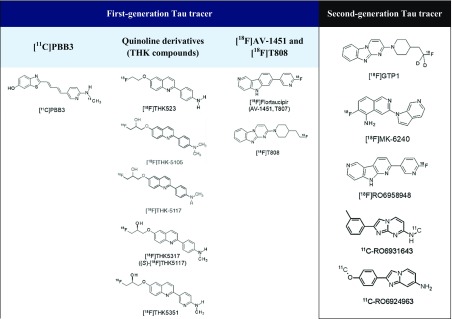
Chemical structures of first and second generation of tau PET tracers

[^18^F]MK-6240, a novel pyridine isoquinoline amine derivative, showed high sensitivity and specificity for PHF-tau binding [57] and displayed favourable kinetics with rapid brain delivery and washout. In AD patients, [^18^F]MK-6240 uptake was higher in brain regions expected to contain NFTs such as the hippocampus, whereas no difference was found in the cerebellar grey matter. It is worth noticing that the cerebellar grey matter showed very low binding across individuals, suggesting the potential for use as a reference region. Reliability analysis revealed robust SUVRs, indicating that simplified quantitative approaches could offer valid estimates of NFTs load [58]. Absence of off-target binding of MK-6240 to MAO-A and MAO-B was confirmed in preclinical studies as well [59]. Recent clinical studies have demonstrated that spatial patterns of MK-6240 binding were consistent with neuropathological staging of NFTs [60]. Unlike [^18^F]T807 and [^18^F]THK5351, off-target binding of MK-6240 was not observed in the basal ganglia and choroid plexus [60]. [^18^F]MK-6240 is a promising tau tracer with the potential to be applied in the disease diagnosis and assessment of therapeutic interventions. To confirm these initial observations, several clinical studies are ongoing in non-AD patients [61]. Another novel tracer [^18^F]PI-2620 has also shown a lack of off-target binding in the choroid plexus, basal ganglia, striatum, amygdala, meninges or other regions noted in first-generation tau agents [62]. In their study, both AD and PSP patients demonstrated evident tracer uptake compared to non-demented controls. Initial pharmacokinetic modelling indicated that SUVr from 60 to 90 min could be a good proxy for DVR, although awaiting validation.

**Fig. 5:**
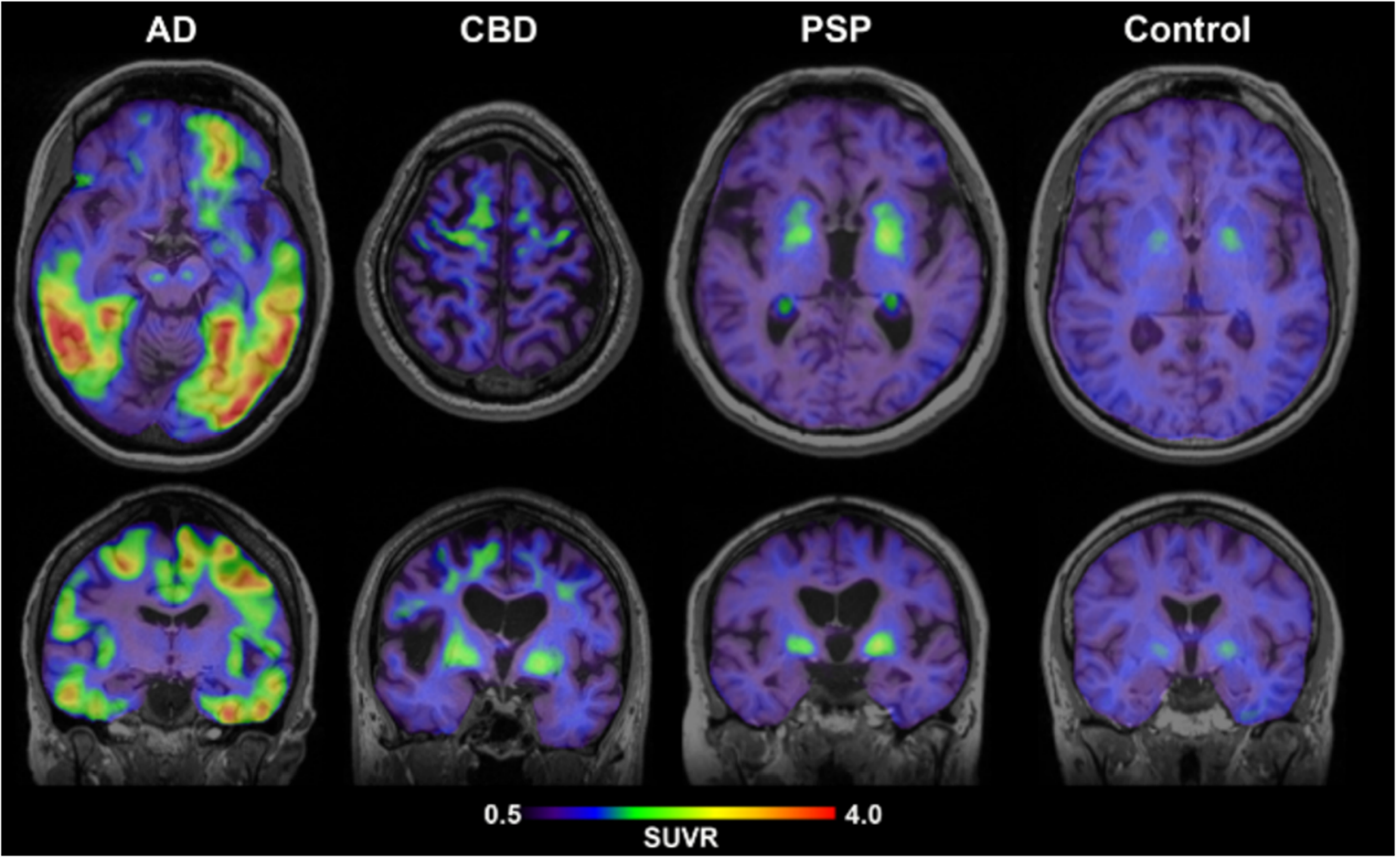
[^18^F]AV-1451 PET images. [^18^F]AV-1451 showed an increased parieto-temporal uptake in an AD subject. Other figures presented the tracer retention in CBD, PSP and a cognitively healthy elderly subject. [Image courtesy of Prof. Oskar Hansson, The Swedish BIOFINDER Study. http://biofinder.se]

**Fig. 6:**
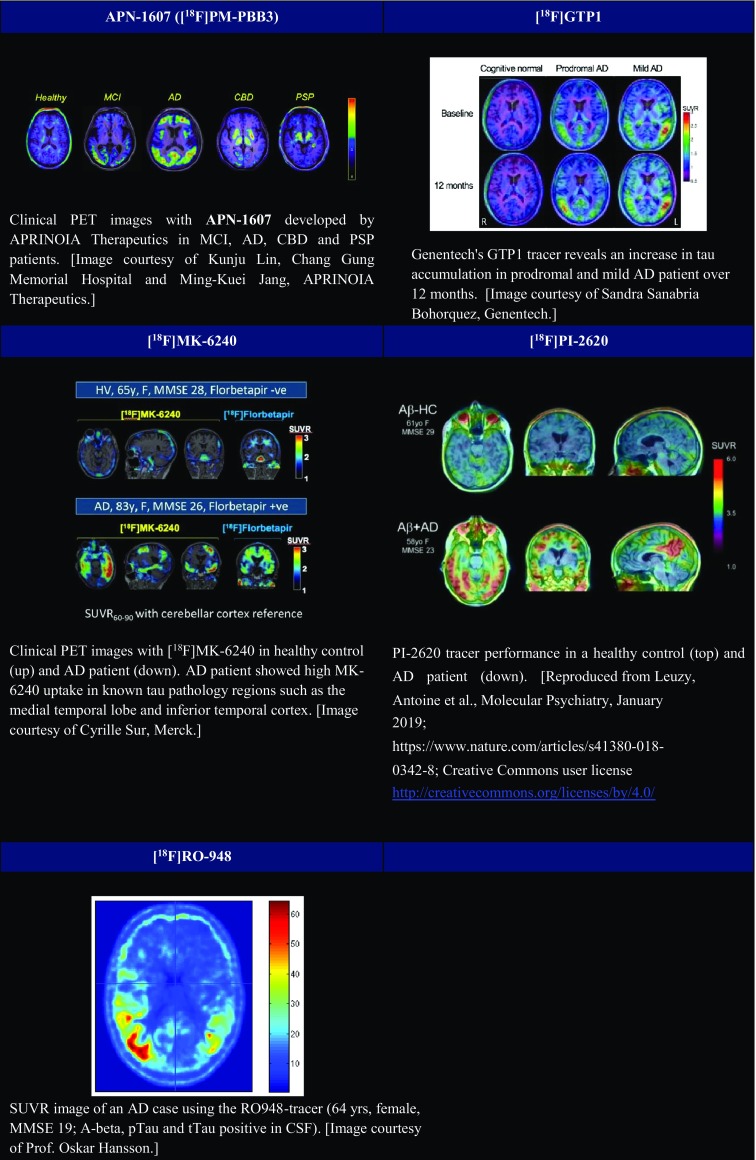
PET images of second-generation Tau tracers. **(**APN-1607 Image courtesy of Kunju Lin, Chang Gung Memorial Hospital and Ming-Kuei Jang, APRINOIA Therapeutics.) (GTP1 Image courtesy of Sandra Sanabria Bohorquez, Genentech) (MK-6240 Image courtesy of Cyrille Sur, Merck.) (PI-2620 Reproduced from Leuzy, Antoine et al., Molecular Psychiatry, January 2019; https://www.nature.com/articles/s41380-018-0342-8; Creative Commons user licence http://creativecommons.org/licenses/by/4.0/) (RO-948 Image courtesy of Prof. Oskar Hansson)

[^18^F]RO6958948 (RO-948), [^11^C]RO6931643 (RO-643) and [^11^C]RO6924963 (RO-963) were identified as high-affinity competitors at the ^3^H-T808 binding site on native tau aggregates in human late-stage AD brain tissue [63]. By macro- and micro-autoradiography and by co-staining of tau aggregates and Aβ plaques on the same tissue section using specific antibodies, the research group was able to assess the uptake, distribution, clearance and metabolism of these three tracers. [^18^F]RO6958948, [^11^C]RO6931643 and [^11^C]RO6924963 all showed good brain entry, rapid washout, high affinity for NFTs and excellent selectivity against Aβ plaques in AD brain tissues. Among them, [^18^F]RO6958948 showed appropriate pharmacokinetic and metabolic properties in mice and non-human primates [63, 64]. Preclinical binding analysis has also proved lower binding affinity of these compounds to MAO-A and MAO-B than the binding affinity of [^18^F]THK5351 and [^18^F]T807. Importantly, the results from a first-in-human PET study were consistent with preclinical data [65•]. In addition, [^18^F]RO6958948 showed a better signal-to-background ratio than [^11^C]RO6931643 and [^11^C]RO6924963 in AD patients [56] (Fig. 5). Clinical PET images and a short summary are given in Fig. 6 and Table 5 respectively.

**Table 5:** Summary of second-generation Tau PET tracers

Tracer	[^18^F]PBB3	[^18^F]GTP1	[^18^F]MK-6240	[^18^F]PI-2620	[^18^F]RO6958948 (RO-948)
					[^11^C]RO6931643 (RO-643)
					[^11^C]RO6924963 (RO-963)
Structural characteristics	^18^F-labelled PBB derivatives		Pyridine isoquinoline amine derivative		
Preclinical study	-Higher signal-to-background ratio	-Rapid pharmacokinetics	-High affinity	-High affinity	-High affinity
	-Less off-target signals than [^11^C]PBB3		-No off-target binding to MAO-A /MAO-B	-High selectivity over Aβ, MAO-A and MAO-B	-High selectivity over Aβ, MAO-A and MAO-B
				-Low off-target binding	
Clinical Human study	-Broader dynamic range compared to [^11^C]PBB3		-Favourable pharmacokinetics	-No off-target binding in regions seen in first-generation tau tracers	-Favourable pharmacokinetics
	-No prominent off-target binding in the basal ganglia and thalamus		-Correlated well with neuropathological staging of NFTs		-High affinity
			-No off-target binding in the basal ganglia and choroid plexus		-High selectivity over Aβ, MAO-A and MAO-B
			-Mild tracer retention in the substantia nigra and meninges		-High signal-to-background ratio
References	[66•]	[56•]	[57•, 59, 60]	[62]	[63, 65•, 67]

### Conclusions and Future Prospects

The advance in in vivo tau imaging has provided exciting and promising results on the usefulness of tau PET in the research of dementia. In vivo tau imaging combined with amyloid and FDG PET imaging could serve as a promising biomarker both clinically and in research. It can support differential diagnosis and the closer association of tau with cognitive impairment as well as neuronal dysfunction makes it suitable for monitoring disease progression. Moreover, it will help identify suitable patients for clinical trials.

Second-generation tau tracers improved substantially in the binding affinity and selectivity compared to first-generation tracers and indeed all these results are leading us onto a new path. However, more in-depth work is still required. Several novel tau tracers are progressing to clinical human studies with results keenly anticipated. In addition, further work involving both tau and Aβ as well as other pathologies, performed at different stages of the disease, will yield more insights into disease pathogenesis. Identification of tau fibril strains accessible to each tau PET ligand would be of critical significance for the use of these tracers to serve as early and differential diagnosis of dementing tauopathies [47•].

In conclusion, tau PET imaging gives a precious opportunity to diagnose dementia accurately, and to evaluate multi-targeted therapy much more efficiently.
